# Characterization of Anti-p54 Monoclonal Antibodies and Their Potential Use for African Swine Fever Virus Diagnosis

**DOI:** 10.3390/pathogens10020178

**Published:** 2021-02-07

**Authors:** Weldu Tesfagaber, Lulu Wang, Ghebremedhin Tsegay, Yibrah Tekle Hagoss, Zhenjiang Zhang, Jiwen Zhang, Haoyue Huangfu, Fei Xi, Fang Li, Encheng Sun, Zhigao Bu, Dongming Zhao

**Affiliations:** 1 State Key Laboratory of Veterinary Biotechnology, National High Containment Facilities for Animal Diseases Control and Prevention, Harbin Veterinary Research Institute, Chinese Academy of Agricultural Sciences, Harbin 150069, China; welduvet@gmail.com (W.T.); wll3118@163.com (L.W.); gherielove84@gmail.com (G.T.); goytomtekle@gmail.com (Y.T.H.); zzj1070303866@163.com (Z.Z.); zjw841380675@outlook.com (J.Z.); haoyue3333@163.com (H.H.); xifei170@163.com (F.X.); lifang01@caas.cn (F.L.); sunencheng@caas.cn (E.S.); 2 Department of Veterinary Science, Hamelmalo Agricultural College, Keren 397, Eritrea

**Keywords:** African swine fever virus, p54, monoclonal antibodies, competitive ELISA, diagnosis

## Abstract

African swine fever (ASF) is a highly lethal hemorrhagic viral disease of domestic pigs caused by African swine fever virus (ASFV). Although a good advance has been made to understand the virus, a safe and effective vaccine against ASFV is still lacking and its eradication solely depends on its early and accurate diagnosis. Thus, improving the available diagnostic assays and adding some validated techniques are useful for a range of serological investigations. The aim of this study was to produce and characterize p54 monoclonal antibodies with an ultimate goal of developing a monoclonal antibody-based enzyme-linked immunosorbent assay (ELISA) for ASFV antibody detection. Five monoclonal antibodies against p54 protein expressed in *Escherichia coli* was generated and their characterizations were investigated. Furthermore, a competitive enzyme-linked immunosorbent assay (cELISA) based on a monoclonal antibody designated as 2A7 was developed. To evaluate the performance of the assay, a total of 365 pig serum samples (178 negative and 187 positive samples) were tested and a receiver-operating characteristic (ROC) analysis was applied to determine the cut-off value. Based on the ROC analysis, the area under the curve (AUC) was 0.982 (95% confidence interval: 96.9% to 99.4%), besides a sensitivity of 92.5% and a specificity of 98.9% was achieved when the percent inhibition of 20% was selected as a threshold. Moreover, the result showed an excellent agreement when compared to other commercially available blocking ELISA (kappa value = 0.912) and showed no reaction to other swine pathogens. Overall, the newly developed cELISA method offers a promising approach for a rapid and convenient ASFV serodiagnosis, which could be used as alternative to other serological assays for screening possible ASFV infection.

## 1. Introduction

African swine fever (ASF) is a highly lethal hemorrhagic viral disease of swine that usually results to a mortality rate approaching 100% in domestic pigs and is classified as a notifiable disease by the World Organization for Animal Health (OIE). Currently, it is the major threat to global pig industry and is caused by African swine fever virus (ASFV), a large and complex double stranded DNA virus with icosahedral morphology [1,2]. Despite of the extensive ongoing research on ASFV, a safe and effective vaccine is still lacking and its control and eradication solely depends on its rapid and accurate diagnosis.

ASFV affects both domestic and wild pigs. However, in domestic pigs there is a variation in the clinical manifestations depending on the virus strains, which differs from an acute, highly lethal hemorrhagic disease to a mild inapparent infection [3,4]. On the contrary, it leads to a mild subclinical infection in the natural reservoir’s hosts (wart hogs and bush pigs), which are the potential source of infection to domestic pigs. It is also important to note that, wild boars (the substantial reservoir of ASFV in Europe and probably also in Asia) show a high susceptibility to ASFV with a disease development similar to domestic pigs [5]. Owing to the importance of the presence of seropositive animals to sub-acute or chronic form of ASFV, there is always a need of an accurate serological diagnosis. Additionally, in the recent years there have been a dramatic increase of ASF outbreak in some Asian and European countries which led to a renewed interest of combating the disease [6,7,8,9]. Therefore, to detect sub-acute or chronic nature of ASF either in domestic pigs or the reservoir hosts, a range of sensitive serological investigations are needed and antibody detection is a rational approach. 

Numerous ELISA-based serological assays integrating the major capsid protein p72/B646L, the structural and highly immunogenic protein p30/CP204L and polyprotein pp62/CP530R antigens are available for ASFV antibody detection [10,11,12,13]. Alternatively, the structural and immunogenic p54/E183L protein is also a highly relevant antigen for serological diagnosis [11,14,15]. P54/E183L is a type II transmembrane protein and contains a potential membrane-spanning domain close to the N-terminus [16]. A two-dimensional gel electrophoresis study on ASFV particle, revealed that p54 has a molecular size of about 25 kDa with an isoelectric point of 6.5 [17]. Also, during cell culture adoption and propagation, ASFV generates virus sup-populations differing in p54 molecular size, which may have a role on the mechanism of genetic diversification of ASFV [16,17]. 

A number of studies have reported that p54 is one of the most important ASFV proteins and plays a key role in virus morphogenesis and viral infection. Anti-p54 sera were found to inhibit ASFV attachment to susceptible cells, suggesting its role in virus entry [18]. Similarly, p54/E183L gene plays an essential role in virus viability [19] and recruitment of envelop precursors to assembly sites [20]. Most importantly, E183L gene is crucial for virus particle transporting to perinuclear factory via direct binding to light chain 8 (LC8) of the dynein [21,22]. A short p54 peptide (149–161 aa) near to its C-terminus is enough to bind to dynein light chain 8 (DLC8) and act as a cargo transport for virus particle [21].

It is proved that, pigs either naturally or experimentally infected with ASFV or inoculated with p54 replicon particle produce high level of anti-p54 antibodies [8,11,15,23,24,25]. Moreover, antibodies against p54 appear as early as 10 days and persist within the blood of infected animals for several weeks [11,15]. All these characteristics in turn makes p54/E183L gene to be one of the best ASFV antigen targets for development of a serological diagnostic assay. In previous studies, ASFV antibody detection using p54 recombinant protein showed 98% sensitivity and 97% specificity on ELISA compared to OIE-ELISA [11] and is a protein of choice for confirmation of ASFV antibody by western blot [14,26]. Although ASFV antibody detection using the most antigenic proteins p72, p30, pp62 together with p54 have been proved to be effective, improving the available diagnostic assays and adding some validated techniques are useful for a range of serological investigations and are a timely demand to mitigate ASFV. Thus, the development of an alternative and more robust diagnostic assay was the focus of this work.

In the current study, a panel of monoclonal antibodies (mAbs) against *E. coli* expressed p54 recombinant protein were generated and their characterizations were investigated. Further assessing on potential use of these anti-p54 monoclonal antibodies as diagnostic reagent for ASFV antibody detection was performed. Subsequently, a competitive-ELISA (cELISA) based on monoclonal antibody designated as 2A7 was developed. The established competitive ELISA showed high diagnostic sensitivity and specificity for ASFV antibody detection. Overall, the work presented here, provides additional evidence with respect to application of p54 monoclonal antibodies for ASFV diagnosis.

## 2. Results

### 2.1. Expression of p54 Recombinant Protein

**Figure 1:**
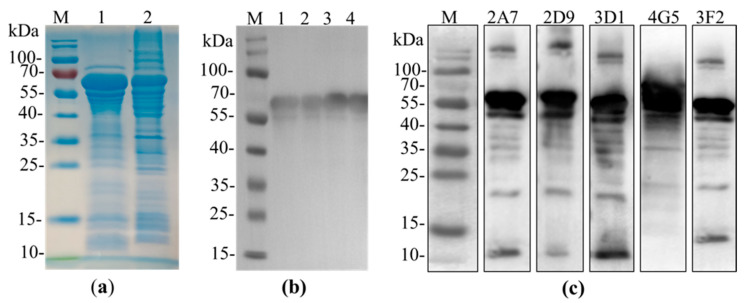
p54 antigen production and analysis of mAbs. (**a**): Sodium Dodecyl Sulfate-Polyacrylamide Gel Electrophoresis (SDS-PAGE) analysis of the Maltose Binding Protein (MBP)-tagged p54 recombinant protein expression, followed Coomassie brilliant blue stain. M is protein marker, lane1 was loaded with 20 µL supernatants of bacterial cell lysates after induction with isopropyl-β-D-1-thiogalactoside (IPTG) and lane2 was loaded with bacterial cell pellets. (**b**): Western blot analysis of purified p54 recombinant protein using anti-MBP tag antibody. All Lane1 to lane4 were loaded with 10 µL purified p54 protein while the left lane is a protein marker. (**c**): western blot analysis for specificity of mAbs, showing all the five p54 monoclonal antibodies recognize the MBP-p54 protein (about 65 kDa).

P54/E183L gene of Pig/HLJ/2018 ASFV isolate was expressed as maltose binding protein-tagged fusion protein in *Escherichia coli* ER2523 strain. The recombinant protein was successfully expressed and following inducing with isopropyl-β-D-1-thiogalactoside (IPTG), an approximate 65kDa fusion protein was observed by sodium dodecyl sulfate-polyacrylamide gel electrophoresis (SDS-PAGE) analysis (Figure 1a). Fusion protein was purified using pre-packed MBP Trap column, confirmed by western blot (Figure 1b) and was used as immunogen to immunize BALB/c mice for monoclonal antibody production.

### 2.2. Anti-p54 Monoclonal Antibody Production

**Figure 2:**
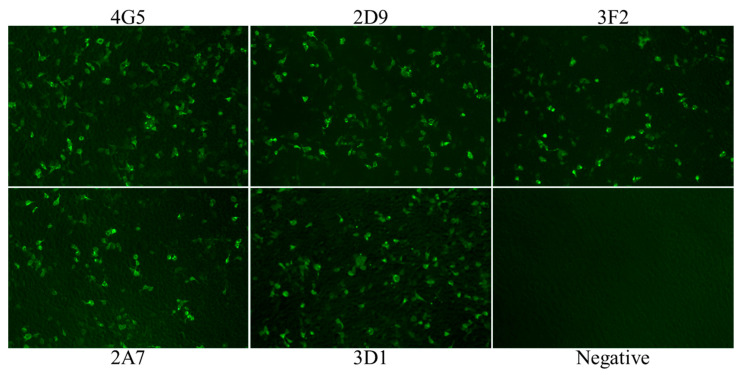
Indirect immunofluorescent assay. HEK 293T cell was transfected with a plasmid expressing viral p54 protein and stained with anti-p54 monoclonal antibodies (4G5, 2D9, 3F2, 2A7 and 3D1) and Alexa Flour 488 FICT- conjugated goat anti-mouse IgG. Following staining all anti-p54 mAbs exhibited green fluorescence. Negative control was HEK 293T cells stained with cell supernatants from SP2/0 mouse myeloma cells.

We set out to produce a panel of monoclonal antibodies against p54 recombinant protein of African swine fever virus. Following the immunization of BALB/c female mice with p54 recombinant protein, mice with a higher antibody titration to p54 was sacrificed and spleen cells were fused with SP2/0 myeloma cells. Eventually, hybridoma were screened by indirect ELISA and five positive clones specific to p54 designated as 2A7, 2D9, 4G5,3F2 and 3D1 were obtained and subcloned three times by a limiting dilution. Isotypes of monoclonal antibodies were characterized using mouse Ig isotyping kit and all were found to be IgG1 with kappa light chain (Table 1). Further screening by Western blot revealed all monoclonal antibodies were able to recognize an approximate 65kDa fusion protein (Figure 1c). Similarly, immunofluorescent assay on HEK 293T cells transfected with p54 recombinant gene was performed and all five anti-p54 monoclonal antibodies were able to react, signifying that produced mAbs were specific to p54/E183L gene of ASFV (Figure 2).

Immunofluorescent assay on ASFV infected peripheral alveolar macrophage cells (PAM cells) was also performed and all mAbs were able to detect ASFV infected cells. No labeling was observed in uninfected cells (data not shown).

### 2.3. Assessing Potential Uses of p54 Monoclonal Antibodies for Competitive ELISA

**Figure 3:**
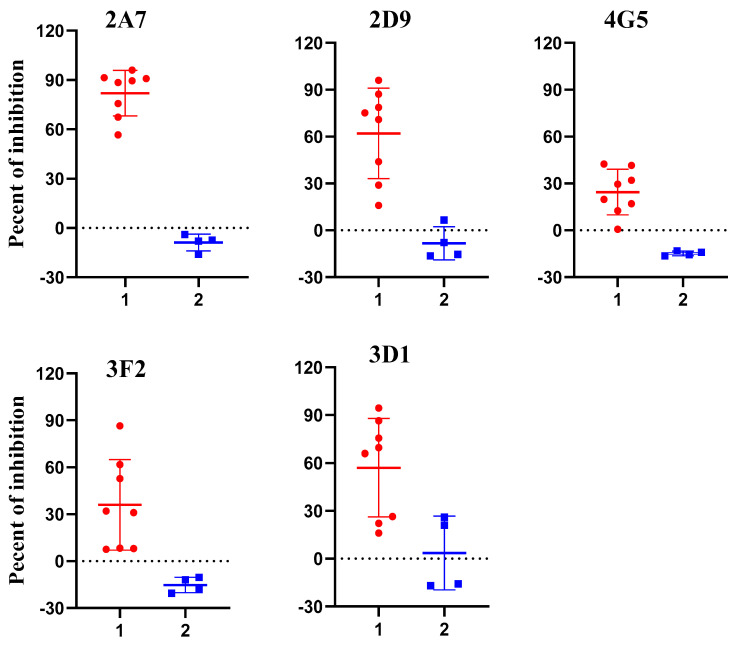
Investigation of p54 monoclonal antibodies on competitive enzyme-linked immunosorbent assay (ELISA) for African swine fever (ASF) detection. In the graph PI value of positive (n = 8) and negative (n = 4) samples are displayed as dots on two vertical axes (1 = positive samples and 2 = negative samples). All positive sera showed PI value greater than 60% when competed with p54 monoclonal antibody designated as 2A7.

To evaluate the potential use of these anti-p54 monoclonal antibodies as a diagnostic reagent for ASFV antibody detection, competitive-ELISA based on each monoclonal antibody was investigated. Eight ASFV positive serum (from experimentally infected pigs) and four negative serum (from specific-pathogen free pigs) were selected to determine which p54 monoclonal antibody will have a good performance to be applied in competitive ELISA. These samples were nominated on the basis of their OD value on indirect ELISA against p54 recombinant antigen. Each sample was tested with a competitive ELISA at a dilution of 1:10 and the percent of inhibition (PI value) of each sample was calculated. The result found on competitive ELISA revealed that all eight positive test samples were able to block mAb-2A7 by greater than 60% with a mean PI value of 82%, while for all the negative samples an inhibition percent of less than zero was recorded when mixed to mAb-2A7 (Figure 3-2A7). Therefore, monoclonal antibody 2A7 was selected to develop a competitive ELISA for ASFV antibody detection.

### 2.4. Optimization of 2A7-Based Competitive ELISA

**Figure 4:**
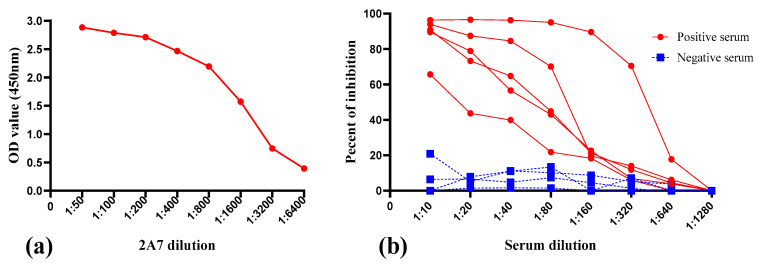
Determination of optimal mAb-2A7 and serum dilution. (**a**): optimization of monoclonal antibody dilution, mAb dilution of 1:1000 was chosen as it consistently produces an OD_450_ value around 1.5. (**b**): determination of optimal dilution of sera. Serial two-fold dilution of five positive and four negative serum samples were tested against a fixed mAb dilution (1:1000) in competitive enzyme-linked immunosorbent assay (cELISA) and percent of inhibition was recorded. For a better inhibition, serum dilution of 1:10 was selected.

Optimal concentration of coating antigen (p54 recombinant protein), mAb-2A7 and test serum samples were determined by checkboard titration procedure. A concentration of 0.2 µg/well of p54 recombinant protein and 1 µg/mL of affinity purified 2A7 were selected as they consistently produce an OD_450_ value around 1.5 (Figure 4a). On the other hand, to optimize serum sample dilution, five known ASFV positive and four known negative serum samples were selected on the basis of their reactiveness to p54 on indirect ELISA. Two-fold serum dilution was tested against a fixed dilution of mAb-2A7(1 µg/mL) and percent of inhibition was calculated. As shown in Figure 4b, the dilution rate of serum samples and the PI value of ASFV positive samples were indirectly proportional with a higher PI value recorded at a dilution of 1:10. On the other hand the PI value of negative serum samples were not influenced with serum dilution rate and were below 20%. Based on this data, serum dilution at 1:10 and 1:20 competes well with 2A7 monoclonal antibody and for an optimal inhibition serum dilution was fixed to 1:10 throughout the experiment.

### 2.5. Standardization and Determining the Negative Cut-off Value for cELISA

**Figure 5:**
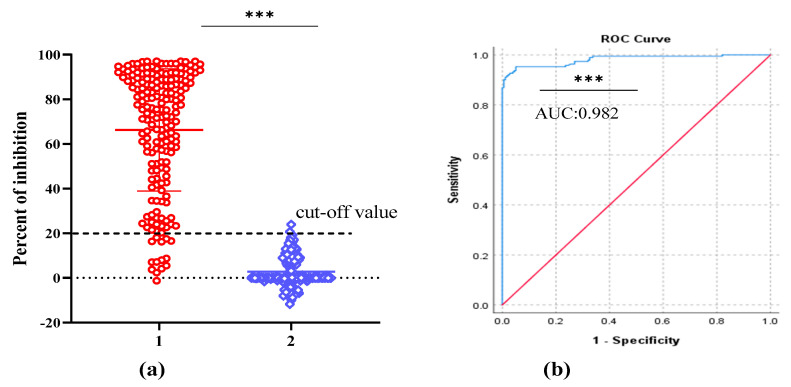
2A7-based competitive ELISA analysis of serum samples. The analysis was performed on known ASFV negative samples (n = 178) and known ASFV positive samples (n = 187). (**a**): interactive dot plot diagram showing the PI value of pig serum samples and cut-off value was set to 20%. In the graph the PI values of positive and negative samples are displayed as dots on two vertical axes (1 = positive samples and 2 = negative samples). (**b**): ROC analysis of 2A7-based cELISA results and the area under the curve (AUC) of the test was 0.982. *p* value was <0.0001 and is indicated by asterisk (***).

After optimizing the protocol for competitive ELISA, a total of 365 pig serum samples (178 negative pig serum and 187 positive samples) were tested to evaluate the performance of the assay. These samples were classified as ASFV seronegative or ASFV seropositive according to their known origin and using a commercial ASFV antibody detection kit (Ingezim PPA COMPAC, Ingenasa, Madrid, Spain). All samples were tested in duplicate by the established cELISA and the percent of inhibition value of each sample was calculated. A receiver operating characteristic (ROC) curve statistical analysis was performed and it allowed us to determine the cut-off value and to estimate the diagnostic sensitivity and specificity of the assay (Figure 5b). In addition, an interactive dot plot diagram outlined the PI value of these samples is shown in Figure 5a. Based on the ROC analysis, the area under the curve (AUC) of the established test was 0.982 (95% confidence interval: 96.9% to 99.4%). Besides, a diagnostic sensitivity of 92.5% (95% confidence interval: 87.8% to 95.9%) and a specificity of 98.9% (95% confidence interval: 96.0% to 99.9%) were achieved, when the cut-off value was set to 20%. By adopting this cut-off value, out of the 187 ASFV positive samples, 173 were correctly placed as ASFV-seropositive, while only two negative samples were diagnosed incorrectly as false positive (Table 2), indicating that the established cELISA has an excellent diagnostic accuracy.

### 2.6. Specificity and Repeatability Test

**Figure 6:**
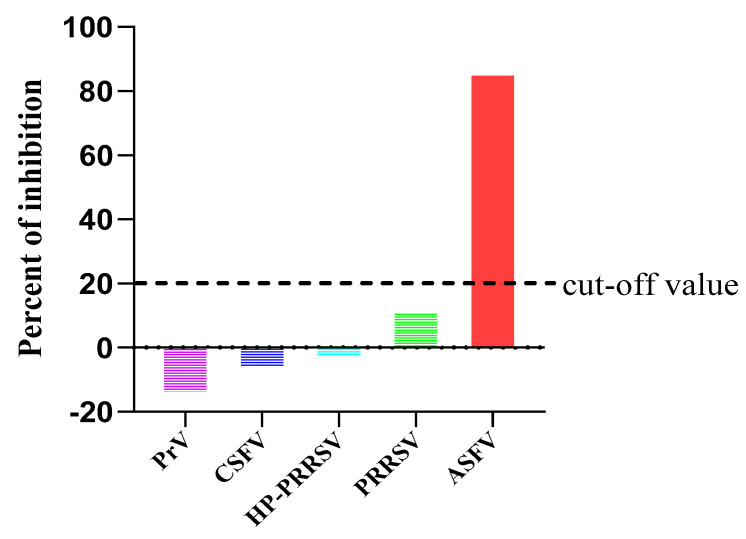
Percent of inhibition of polyclonal anti sera against various porcine viruses after detection with 2A7-cELISA. Only the ASFV positive pig sera had a PI value exceeding the cut-off value.

To confirm its specificity, the developed cELISA was used to detect four polyclonal anti-sera against other swine viruses. All sera yielded a negative result in the competitive ELISA, with a PI value much lower than the cut-off value (Figure 6). Thus, non-specific positive swine sera were clearly discriminated from the ASFV positive sera, suggesting that the established cELISA has a satisfactory analytical specificity.

To evaluate repeatability of the cELISA, ten randomly picked serum samples (five positive and five negative) were tested by the developed cELISA and the intra and inter-assay variation was determined by calculating the coefficient of variation (CV%). For intra-assay variation, OD values of those ten samples were measured on triplicate on the same plate, while, for inter-assay variation OD values of same samples were measured three times on ELISA plates coated at different times. According to Jacobson [27] an assay with a coefficient of variation (CV) less than 20% for raw absorbance value is considered to have an adequate repeatability. In this study, the result produced an intra-assay CVs ranging from 0.78% to 11.27% and an inter-assay CVs ranging from 5.79% to 16.8%, showing an excellent repeatability (Table 3).

## 3. Discussion

In the past 100 years, ASFV has emerged as a major threat to global pig industry. According to the World Organization for Animal Health report on June 2020, the disease is currently circulating in many Asian and European countries, namely, Moldova, Czech Republic, Romania, Hungary, Bulgaria, Belgium, Slovakia, Serbia and Greece from Europe and China, Mongolia, Vietnam, Cambodia, Korea, Laos, Myanmar, Philippines, Indonesia, Papua New Guinea and India from Asia [9]. Equally, it is causing a serious deterioration and incalculable economic impact due to its fast spread.

ASFV virion is a complex, multilayered structure with overall icosahedral morphology and encodes a large number of polypeptides [1,28]. Besides, the incomplete understanding of the complex viral structure and its immuno-determinant viral proteins hinders vaccine development against ASFV [29], which has made control and eradication of ASF to depend on applying strict quarantine and biosecurity measures, restriction of animal movements and slaughtering of affected animals [6]. Moreover, to apply these control and eradication measures, an early and accurate diagnosis of ASFV is valuable. On this regard, diagnostic identification of antibodies is a major epidemiological tool used to detect the occurrence of ASFV infection, where clinical signs have not been manifested. For instance, presence of ASFV antibodies implies previous infection and are good markers for disease diagnosis [11,30]. Moreover, antibodies against ASFV appears soon after infection and persist for several months to years [31]. Therefore, a sensitive and reliable serological diagnostic assay is required, so as laboratories can effectively and quickly detect ASFV infection. Corresponding to this, identifying potential antigenic ASFV protein targets that suits to develop a diagnostic assay is very important and the structural p54/E183L protein along with p72, p30 and pp62 are among the most.

In the current study, we generated five monoclonal antibodies against the *E. coli* expressed p54/E183L protein of ASFV and their diagnostic application were evaluated. Analysis of those mAbs with ELISA, western blot and IFA showed that all five monoclonal antibodies were able to recognize the immunizing p54/E183L antigen. The structural p54 protein was fused to MBP in our study and results in a protein with a higher molecular mass about 65kDa, when compared to the actual predicted molecular mass of p54/E183L (25 kDa) [17]. To develop a sensitive and reliable cELISA for ASFV antibody detection, the five mAbs were examined for their ability to compete with polyclonal antibodies from ASFV infected pigs. Accordingly, a monoclonal antibody based-competitive ELISA (2A7-cELISA) was developed. Monoclonal antibody 2A7 was selected, because it demonstrates the strongest competition with ASFV-seropositive samples but not with negative sera (Figure 3).

Generally, true negative and true positive samples defined by a “gold standard” method are very important to calculate the diagnostic specificity and sensitivity of a newly established assay. However, as gold standard is difficult to achieve, relative standards of comparison either from experimentally infected or vaccinated animals is often necessary [27]. For this purpose, 187 sera from pigs with a known exposure to ASFV infection and 178 sera from pigs that do not have any history of contact to ASFV were used in our experiment. Similarly, a commercially available blocking ELISA kit (Ingezim PPA COMPAC) for ASFV antibody detection were used as a standard evaluating method. The ASFV Ingezim PPA COMPAC is OIE recommended commercially available blocking ELISA [32] and is one of the three commercial ELISA kits in use by the Foreign Animal Disease Diagnostic Laboratory (FADDL) [11]. Initially, serum samples were classified as ASFV-seronegative and ASFV-seropositive based on their origin and their result on Ingezim PPA COMPAC and later all the samples were tested by the newly developed cELISA. Considering the PI value of these 365 pig serum samples, a receiver-operating characteristic (ROC) analysis was used to estimate the accuracy of 2A7-based cELISA. According to the result from ROC curve approach, nearly 100% diagnostic specificity was obtained, when the cut-off value was set to 25% but the sensitivity of the test was 86.6%. Thus, we select a cut-off value of 20%, which presents the optimal balance of sensitivity (92.5%) and specificity (98.8%). Also, the result achieved from the newly developed cELISA showed an excellent agreement (kappa value = 0.912) with those obtained from Ingezim PPA COMPAC, which indicates a very good concordance between the two methods.

To our knowledge, the two commercially available blocking-ELISA for ASFV antibody detection depends on the use of monoclonal antibodies against p72 (Ingenasa-Ingezim PPA COMPAC K3; Ingenasa, Madrid, Spain) and p30 (ID.vet- blocking ELISA; ID Screen African Swine Fever Competition, Grabels, France). This work identifies the usefulness of p54 monoclonal antibodies on this regard and will extend the previously available cELISAs for the diagnosis of ASFV infection. As a whole, the developed cELISA based on p54 monoclonal antibody has a high diagnostic sensitivity and specificity. Although, further validation using large sample size is strongly recommended, the work presented here encourages the use of p54 monoclonal antibodies in competitive ELISA for ASFV antibody detection.

## 4. Materials and Methods

### 4.1. Serum Samples

A panel of 365 serum samples were analysed with the established 2A7-based competitive ELISA, including 178 negative sera and 187 ASFV positive sera. The origin of samples was:

(A) 178 samples without ASFV exposure: out of these 148 field samples were collected in 2017, before the outbreak of ASFV in China and were confirmed to be negative by the commercial ASFV antibody detection kit, while the other 30 negative sera were originated from specific pathogen free (SPF) pigs.

(B) 187 samples with ASFV exposure: all ASFV positive samples used in this study were obtained from pigs experimentally inoculated either with the wild type Pig/HLJ/2018 ASFV isolate or with the recently developed seven-gene deleted ASFV vaccine candidate HLJ/18-7GD [33]. Those samples were collected at different time of interval between the year 2019 to 2020 in our laboratory and their status was determined by the commercial ASFV antibody detection kit.

(C) Standard control: hyperimmune serum from pig immunized with p54 recombinant protein was used as a positive control.

(D) Heterologous positive sera: to evaluate the specificity of the assay, four known positive sera for other porcine pathogens, namely, porcine reproductive and respiratory syndrome (PRRS), highly pathogenic porcine reproductive and respiratory syndrome (HP-PRRSV), Classical swine fever virus (CSFV) and Pseudorabies virus (PrV) were tested. All anti-sera were detected and certified by Harbin Guo sheng Biological Testing Technology Co., Ltd, Harbin, China.

### 4.2. Constructing p54 Recombinant DNA

P54/E183L (excluding the predicted transmembrane domain) based on African swine fever virus isolate Pig/HLJ/2018 (accession. No. MK333180.1) [34,35] was amplified using a forward primer TCTTCAAGAAAGAAAAAAGCTGCTG and a reverse TTACAAGGAGTTTTCTAGGTCTTTATGCGT. Amplicon were inserted downstream from the *malE* gene of *E. coli* into the vector pMAL-c5x using *NdeI* and *EcoRI* restrictions sites introduced into the sense and antisense primers, respectively. Briefly, polymerase chain reaction (PCR) amplified E183L/p54gene (399bp) and pMAL-c5x vector were digested with *NdeI* and *EcoRI* restriction enzymes and accordingly ligated with T4 DNA ligase. Recombinant gene (pMAL-c5x-54) were then transformed to DH5α *E. coli* competent cells and incubated over night at 37 °C in an agar plate with ampicillin. On the next day, perfection of the correct insert was checked by PCR and positive samples were confirmed by DNA sequencing (Comate Bioscience Co., Ltd, Jilin, China).

### 4.3. Expression and Purification of Recombinant ASFV-p54 Protein

Recombinant E183L/p54 gene of ASFV isolate Pig/HLJ/2018 was expressed as maltose binding protein -tagged (MBP-tagged) fusion protein in *Escheria coli* ER2523 strain (made competent in our laboratory according to a protocol by Chung [36]). Expression was facilitated by adding 0.5 mM isopropyl-β-D-1-thiogalactoside (IPTG) and successful expression was examined by sodium dodecyl sulfate-polyacrylamide gel electrophoresis (SDS-PAGE) analysis of cell lysates. To purify p54 recombinant protein, bacterial cells were harvested by centrifugation, resuspended in pre-cold PBS (25 mL/liter of bacterial culture) and lysed by sonication. After centrifugation at 12,000 rpm for 30 minutes, supernatants were collected and filtered through 0.22 µm and purified using pre-packed MBP Trap column, operated by AKTA AVANT liquid chromatography system. Buffers for purification were prepared according to the manufacturers guide (binding buffer = 20 mM Tris-HCl and elution buffer 10 mM maltose in binding buffer).

### 4.4. P54 Monoclonal Antibody Production

To produce anti-p54 monoclonal antibodies the standard protocols for hybridoma technology was followed [37]. Female mice (BALB/c), 4–6 weeks age, were immunized by intraperitoneal injection with 100 µg of purified p54 recombinant protein, which was emulsified with an equal amount of Freund’s complete adjuvant (Sigma, Lot # SLBZ9888, St. Louis, MO, USA). A conventional immunization protocol was followed on three mice, to select the mouse best responding to p54 antigen. Two booster immunization was done at two-week intervals mixed with incomplete adjuvant. 10 days later from the second boost, blood was taken by tail bleeding and serum antibody titration for p54 was determined. Mouse that presented the highest antibody titration by indirect ELISA was chosen for the final boost without adjuvant and subsequently was sacrificed three days later to harvest spleen cells. 

Hybridoma were obtained by fusing SP2/0 mouse myeloma cells with spleen cells by using poly ethyl glycol (PEG) (Sigma, Lot # RNBF1747, St. Louis, MO, USA). Fused spleen and SP2/0 cells were resuspended with RPMI-1640 medium (Gibco, Lot # 8120219, ThermoFisher Biomedical products Co., Ltd, Beijing, China) supplemented with 20% FBS, 1% antibiotic (penicillin and streptomycin), 0.2% glutamine and 50× hypoxanthine-aminopterin-thymidine (HAT) (Sigma, Lot # SLBZ5521, St. Louis, MO, USA). Thereafter, fused cells were dispensed to 96 well micro culture plate (100 µL/well), added to previously prepared feeder cells and incubated under 5% CO_2_ at 37 °C. Feeder cells used were BALB/c macrophages, harvested from healthy BALB/c mice by peritoneal wash with 20% FBS-RPMI medium, distributed in to four 96 well micro culture plate and incubated for 24 hours prior to fusion. After five days of culture, growth medium was replaced with medium containing 50x HAT and on day seven medium containing only 50x hypoxanthine-thymidine (Sigma, Lot # SLBX7957, St. Louis, MO, USA), was used to replace the growth medium in order to reduce the effect of aminopterin. Cell supernatants were assayed 10 days post cell fusion and wells with confluent hybridomas were screened by indirect ELISA using p54 recombinant protein as a coating antigen. subsequently, positive colonies were selected and sub-cloned by limiting dilution to obtain a single hybrid cell per well. 

### 4.5. Western Blot Analysis

The reactivity of monoclonal antibodies to p54 recombinant protein was examined by western blot analysis. Purified MBP-tagged p54 recombinant protein was separated in a 12% SDS-PAGE by electrophoresis and separated proteins were transferred in to PVDF membrane. The membrane was then blocked for 1 hour with 5% skimmed milk. Primary antibodies were added and incubated for 2hrs at room temperature with a slow agitation. The primary antibodies used were, five anti-p54 monoclonal antibodies (undiluted hybridoma supernatants), anti-MBP monoclonal antibodies diluted as 1:10,000, polyclonal mice sera from mice immunized with recombinant p54 protein diluted 1:400. After extensive washing with PBST, HRP-labelled goat anti-mouse IgG was added and left at room temperature for 1 hour. Finally, protein bands were visualized by digital imaging system after three-time wash with PBST.

### 4.6. Indirect ELISA

For the screening of antibody secreting hybridoma cells, standard protocol for indirect enzyme-linked immunosorbent assay was performed [38]. Briefly, ninety-six-well microtiter ELISA plates were coated with 0.2 µg recombinant p54 protein per well (coating concentration was determined by checkboard titration) and incubated overnight at 4 °C. The plate was washed five times with PBST (0.05% Tween in PBS, *v*/*v*) and plate was blocked with 5% skimmed milk in PBS, for 1 h at 37 °C. After washing the plates as above, 50 µL undiluted hybridoma supernatants were added. Positive sera from mice immunized with p54 recombinant protein and negative sera from unimmunized mice, diluted 1:200 was also included in duplicate as a control. The plate was incubated for two hours at 37 °C and washing step was repeated. Thereafter, horse radish peroxidase (HRP) conjugated goat anti-mouse IgG diluted 1:10,000 was added and incubated for1 h at 37 °C. Following extensive washing, reaction was developed by adding chromogenic substrate solution (TMB) for 10 minutes and stopped with 2M sulphuric acid. Result was read at OD_450_ absorbance.

### 4.7. Indirect Immunofluorescent Assay (IFA)

To evaluate the specificity and ability of anti-p54 monoclonal antibodies to detect ASFV infected cells, IFA was performed on HEK293T cells transfected with p54 recombinant gene and ASFV infected PAM cells. HEK293T cells were grown in 24-well plate at a density of 1.25 × 10^5^/well in a complete DMEM medium. After 24 hours, cells were transfected with p54 plasmid DNA and incubated at 37 °C in 5% CO_2_. 24 hours post transfection, HEK293T cells were washed with PBS and fixed with 4% formaldehyde for 10 minutes at room temperature. Following washing with PBS, 0.25% triton was added to each well in order to increase the permeability of the cells and was left at room temperature for 10 minutes. The cells were then incubated with anti-p54 monoclonal antibodies for 1 hour at 37 °C, followed with FICT conjugated goat ant-mouse IgG. After each incubation step, the wells were washed four times with PBS. Finally, result was observed using a fluorescent microscope.

IFA was also done on ASFV infected peripheral alveolar macrophage cells (PAM cells). The general procedure was same with IFA done on HEK 293T cells, except PAM cells were grown in complete RPMI medium supplemented with 10% porcine serum and were infected with the lethal dose of ASF virus (Pig/HLJ/2018 isolate).

### 4.8. Establishment of Monoclonal Antibody Based-Competitive ELISA for African Swine Fever Antibody Detection

Monoclonal antibodies based-competitive ELISA was performed to assess the potential application of p54 monoclonal antibodies for ASFV diagnosis. The general procedures followed was as follows; ELISA plates were coated with 0.2 µg/well p54 antigen and incubated over night at 4 °C. The optimal coating concentration of recombinant protein and dilution of monoclonal antibodies (mAbs) was determined by checkboard titration. On the next day, plates were washed five times with PBST, blocked with 200 µL/well 5% skimmed milk and incubated for 1 hour at 37 °C. Following five times wash, plates were either used directly or kept in −20 °C for further use. To optimize serum dilution, a serial two-fold dilution (1:10, 1:20, 1:40, 1:80, 1:160, 1:320, 1:640 and 1:1280) were tested against a fixed monoclonal antibody dilution. Later, each well of p54 antigen coated plates were incubated with 50 µL pig serum diluted with PBS for 30 minutes at 37 °C. An equal amount of p54 monoclonal antibodies (50 µL/well) diluted in PBS was then added to each well without being washed. Controls consisting of wells without mAb or pig serum and wells having only mAb were included. Serum and mAb mixture were incubated for 1hr at 37 °C. Washing step was repeated and HRP conjugated goat anti-mouse IgG (ZSGB-Bio, China, catalog # ZB-5305) was added and incubated for 1 hour at 37 °C. After extensive washing, 50 µL color substrate (TMB) was added to each well and left at room temperature for 15 minutes and reaction was terminated by adding 50 µL 2M sulphuric acid to each well. Finally, result was read at optical density of 450 nm. The raw data was transformed to an excel spread sheet and accordingly the percent of inhibition (PI value) of each test sample was calculated using the formula: PI (%) = [(OD_450_ value of negative controls − OD_450_ value of sample)/OD_450_ value of negative controls] × 100%, as described by Wang and his colleagues [39].

### 4.9. Data Analysis

Using the commercial blocking ELISA kit as a standard evaluating method, sensitivity and specificity of the established cELISA was calculated by the web-based MedCalc statistical software (https://www.medcalc.org/calc/diagnostic test.php (accessed on 7 February 2021)). Receiver operating characteristic (ROC) analysis and degree of agreement (kappa value) were analysed using SPSS software for windows, version 26.0 (IBM, Armonk, NY, USA). Similarly, Interactive dot plot diagram was performed via Graph pad prism version 8.0.2. (GraphPad Software Inc. La Jolla, CA, USA) and difference were considered statistically significant when *p* value was less than 0.05.

## 5. Conclusions 

This study prepared five monoclonal antibodies against the structural p54 protein of ASFV and their diagnostic application were investigated. Through the aforementioned experiments and analysis, we conclude that the newly developed mAb 2A7 based cELISA method offers a promising approach for a rapid and convenient ASFV serodiagnosis, which could be used as alternative to other serological assays for screening possible ASFV infection. 

## Figures and Tables

**Table 1:** Identification of subclasses of p54 monoclonal antibodies.

	Monoclonal Antibodies				
	2A7	2D9	4G5	3F2	3D3
Ig subclass	IgG1	IgG1	IgG1	IgG1	IgG1
Light chain type	κ	κ	κ	κ	κ

**Table 2:** Relative sensitivity and specificity of the newly established cELISA.

Ingezim PPA COMPAC Result	Serum Samples Tested with P54-cELISA		
	Positive	Negative	Total
Positive	173	14	187
Negative	2	176	178
Total	175	190	365

Relative sensitivity = 173 of 187 or 92.5%, relative specificity = 176 of 178 or 98.9%.

**Table 3:** Intra and inter-assay repeatability of the newly developed cELISA.

Samples	Intra-Assay			Inter-Assay		
	Mean OD Value	SD	CV%	Mean OD Value	SD	CV%
Positive 1	0.090	0.003	**2.78**	0.0903	0.0103	**11.36**
Positive 2	0.082	0.008	**9.52**	0.069	0.006	**8.07**
Positive 3	0.072	0.008	**11.27**	0.057	0.005	**8.77**
Positive 4	0.099	0.004	**4.09**	0.101	0.013	**12.98**
Positive 5	0.150	0.004	**2.40**	0.137	0.023	**16.80**
Negative 1	0.915	0.033	**3.60**	0.95	0.09	**9.67**
Negative 2	0.945	0.007	**0.78**	0.89	0.05	**5.79**
Negative 3	0.964	0.067	**6.92**	0.96	0.14	**14.04**
Negative 4	0.927	0.016	**1.67**	1.04	0.06	**5.91**
Negative 5	0.924	0.053	**5.72**	0.82	0.07	**8.79**

## Data Availability

Data sharing is not applicable to this article.
